# Account of an Extraordinary Change, Not Hitherto Described, Which, under Certain Circumstances, Takes Place in the Human Body after Death

**Published:** 1791

**Authors:** M. Thouret


XIX. *Account of an extraordinary Change, not hitherto defcribed, which, under certain Circumftances, takes place in the human Body after Death.*—Vide *Rapport fur les Exhumations du Cimetiere et de l'Eglife des Saints Innocens; lû dans la Seance de la Société Royale de Medecine tenue au Louvre le 3 Mars, 1789. Par M.* Thouret. 4to. Paris, 1790.

IN this report, relative to the removal of the bodies from the church and church-yard of the Holy Innocents, M. Thouret, who is a very refpectable phyfician at Paris, and already well known to the Public by his writings, gives an account of a very extraordinary change to which the human body, under certain circumftances, is fubject after death.

The fituation of the burial place in queftion, in the center of the city of Paris, has for a great length of time pointed it out as a nuifance to the Public. Its fuppofed unhealthinefs occafioned it to be a fubject of inquiry fo long ago as the year 1557, when two phyficians, Fernelius and Houllier, were directed by Government to examine it; and in 1737 a Committee of the Academy of Sciences was appointed pointed for the fame purpofe. On both thefe occafions the removal of it was earneftly recommended; but it does not appear that any fteps were taken to remedy the inconvenience complained of till the year 1780, when an order was iffued to prevent any more burials in this fpot. This regulation, however, M. Thouret obferves, which might have been fufficient in the generality of places of this kind, where the bodies, being but thinly interfperfed in the earth, are fpeedily deftroyed, was altogether inadequate to the evil in the prefent inftance, the foil being here fo faturated with animal matter as to be no longer capable of any action on the more recent bodies accumulated within it.

M. Thouret obferves, that fince the year 1186 this place has ferved as a common burial place for the greater part of the city of Paris, and that for a great number of years paft from two thoufand five hundred to three thoufand bodies have been interred in it annually. He has been affured, it feems, that in a fomewhat lefs fpace of time than thirty years upwards of eighty thoufand bodies were interred in it by the laft fexton. This immenfe collection of dead bodies occupied, we are told, a furface of more than ten thoufand fquare feet. They were accumulated, for the moft part, in common graves, or pits, from twenty five to thirty feet deep, each of which was large enough to contain from twelve to fifteen hundred coffins: and as a proof how few bodies were buried in feparate graves, we are told that the number of fuch interments feldom exceeded two hundred annually.

At length, Government having determined to remove this nuifance, the Royal Medical Society were called upon to point out the beft mode of doing it; and our author, who was one of the Committee appointed by the Society for that purpofe, and who fuperintended the whole of the undertaking, now communicates the refult of his obfervations on this fubject to the Public. The operations lafted upwards of two years, and during that period, it feems, a layer of earth from eight to ten feet deep was removed from the furface of the burial ground to the extent of twelve thoufand fquare feet, and, befides a great number of feparate graves, between forty and fifty of the common receptacles were opened to the depth of eight or ten feet, and fome of them to their very bottom, and and about twenty thoufand bodies, buried at different periods, were removed with their coffins.

Amidft a great variety of appearances which fo many bodies exhibited, from their having been interred a greater or lefs fpace of time, in feparate graves or in the common receptacles, there was one extraordinary circumftance which foon ftruck our author's attention. This was the ftate of the coffins and bodies in the common pits. The coffins in thefe were, in general, firm and in good prefervation; and the earth that furrounded them was of a deep black colour; but, excepting this blacknefs which had tinged the coffins externally, they retained their frefhnefs, and withinfide the natural colour of the wood was eafily diftinguifhable. The fhrouds were obferved to be in the fame ftate of prefervation, and the bodies themfelves appeared to be undiminifhed in bulk. Upon removing the fhroud the flefhy parts of the bodies feemed to be preferved; the only change that was perceived confifted in their being converted, as it were, into a fubftance, the whitenefs of which was heightened by the blacknefs of the furrounding foil.

The author tells us that at firft fight of this curious phenomenon he was inclined to confider fider it as the effect of lime fpread over thefe bodies; but upon examining them more attentively he was foon convinced that he was wrong in this fuppofition, and he found that all the foft parts were converted into a white mafs, more or lefs firm, and already known among the gravediggers by the name of fat, *(gras.)* This mafs, which exhibited no appearance of a fibrous texture, felt unctuous or foapy when rubbed between the fingers, and in a dry air grew harder, and even acquired a fhining polifh and a fort of metallic luftre, but became fofter when expofed to a moift air.

In general thefe maffes, the author obferves, preferve the entire fhape of the limbs. Among the bodies which he found the moft completely transformed into this fubftance, and which form a part of the collection he has made to illuftrate the hiftory of this phenomenon, feveral, he tells us, retain their natural fhape, together with the features of the face, the eyes, eyebrows, and eyelids. The tranfmutation, he obferves, is not confined to the furface of the body, but may be traced through every part of the mufcles, ligaments, and tendons, and likewife through the different cavities, where all or the greater part of the vifcera are found converted verted into the ſame ſubſtance, which is alſo to be ſeen in the cavities of the bones, even in the cells of the diploe. It is found to affect the texture of the cartilages, but the bones themſelves, it ſeems, remain unaltered, as do likewiſe the hair and nails. There are likewiſe, we are told, certain colouring principles, ſuch as the bile, the fluid of the bronchial glands, the pigmentum of the choroid, the red particles of the blood, and the fibrous part of the muſcles, which remain for a long time diſtinguiſhable in the maſs that ſurrounds them.

The parts that have appeared to our author to be the moſt ſuſceptible of this change have been the adipoſe and membranous. Some parts, he obſerves, evidently acquire it much ſooner than others, and he has found the blood veſſels of different viſcera, particularly thoſe of the liver, transformed into this maſs, while the ſurrounding ſubſtance of the viſcus itſelf had as yet undergone no ſuch change.

He obſerves that, in general, the parts preſerve their natural configuration in proportion to the quantity of adipoſe and lymphatic juices they contain, and in proportion to the denſity of their texture. Thus the brain, the heart, the liver, and ſome other viſcera, it ſeems, change change completely into this ſubſtance, and retain their original figure, while of the inteſtines and the ſpongy and veſicular texture of the lungs only ſlight veſtiges remain after this change, and in theſe the fatty ſubſtance into which they are converted is of a much thinner conſiſtence than in the other parts.

From a chemical analyſis of this ſubſtance, for which our author acknowledges himſelf indebted to M. Fourcroy, it appears to conſiſt of an oily principle combined with volatile alkali ſo as to form a ſoap. The oily baſis of this ammoniacal ſoap, ſeparated by acids, is deſcribed as a concrete ſubſtance, of a grayiſh yellow colour, and ſomewhat more fuſible than wax; combined with fixed or volatile alkali it forms, we are told, a firm ſoap.

M. Thouret remarks, that it is not ductile under the fingers like wax, but that it crumbles into ſmall, ſoft, and unctuous fragments like ſpermaceti, the ſubſtance with which he conſiders it as having the greateſt analogy. Thus he obſerves that it chryſtalliſes like ſpermaceti, and diſſolves even in a greater proportion than that does in heated alcohol; part of it ſeparating again as the ſolution cools, in the form of ſmall ſhining laminæ.

From theſe data our author is led to attempt a theory of the formation of this ſubſtance. He aſcribes it to a peculiar modification of the putrid change that bodies undergo in the earth, and thinks that the origin of all the phenomena is to be ſought for in the decompoſition of water. It has been ſuppoſed, he obſerves, that from a combination of phlogiſticated with inflammable air there reſults, during putrefaction, volatile alkali; and the fixation of a larger proportion of inflammable air, and perhaps alſo of a certain quantity of dephlogiſticated air, may, he thinks, give riſe to a fat or oily ſubſtance, which, by uniting with the volatile alkali, forms a ſoap.

M. Thouret obſerves, that a concretion analogous to this ſubſtance is not foreign to the living animal œconomy; that it exiſts, as is well known, in large maſſes in the cavities of the brain of the whale, and is diſtributed by numerous veſſels through all the parts of that animal; and that it is alſo to be found in the bile, where till of late it has been taken for a reſin. It has ſometimes, he adds, been found extravaſated in the liver when dried in the air, as was proved by the late M. Poulletier de la Salle, of Paris, who, having expoſed a human liver to the air for a confiderable number of years, found it changed, at length, into a whitifh mafs, in its appearance not unlike agaric, and which, on expofure to a gentle heat, yielded a fubftance fimilar to fpermaceti. M. Thouret affures us his experiments have taught him that a fubftance of the fame kind may be extracted in abundance from the brain of man and other animals. May it not, therefore, he afks, be latent in the living body, and intended to anfwer fome purpofe in the animal œconomy with which we are as yet unacquainted?

This fingular tranfmutation, he obferves, though it is found to affect bodies of both fexes, and of all ages, is fubject, however, to fome differences which have not efcaped the notice of the gravediggers, who have remarked that bodies which are the fatteft and moft compact pafs the fooneft into this ftate; that very dry and lean ones acquire more of the appearance of dry mummies; and that lax and humid ones melt into water.

The tranfmutation, whatever may be its nature, takes place, we are told, indifferently in different kinds of earth. It likewife appears to be completed in a fhort fpace of time. The laft laſt great pits of the burial place had been cloſed, it ſeems, only five years, and from the ſurface to the bottom all the bodies they contained, a very ſmall number excepted, were found by our author transformed into the ſubſtance in queſtion.

In general, however, the manner in which this tranſmutation, when once begun, goes on and is completed, appears to be not altogether uniform. In the pits where it ſeemed to be the moſt completely effected, the greater number of bodies, we are told, were entirely transformed; but, on the other hand, in ſome the change appeared to be only juſt beginning to take place, while in others the decompoſition was complete. In the ſmall number that afforded no marks of it the bones only remained, and theſe exhibited the common appearance. Were theſe, the author aſks, the remains of bodies that had paſſed through this ſtate, and had afterwards been totally deſtroyed? There was nothing, he obſerves, in the ſituation of theſe laſt that could explain the difference. They were found at all depths, and cloſe to others in which the change was complete. In general, however, it ſeems, it was in the bodies at the greateſt depth that the change appeared to take place

 the the fooneft, and thefe alfo feemed to be the laft in which this fatty fubftance was deftroyed. Our author found this fact confirmed by what he faw in two other burial grounds at Paris.

It appears from M. Thouret's obfervations, that the fkin is the part in which this change firft begins to take place, and that after this follow the fat, the mufcles, and the vifcera. In the early ftage of the tranfmutation the texture of the fkin, we are told, is ftill diftinguifhable, as is alfo the colour of the fat and of the mufcles, and it is not till the fibrous texture of the latter has entirely difappeared that the change can be faid to be complete. When this is accomplifhed, a decompofition begins to take place. This is firft obfervable in the cavities of the body, and as it advances the bones become difunited, the fatty fubftance is gradually diffolved, and at length there remain only flight appearances of it adhering to the furface of the bones; but in this ftate it has the confiftence and colour of clay, or becomes dry and friable and of a darker colour. M. Thouret fuppofes this to be the remains of the colouring principle, or of the earthy principle ftill combined with a little of the fatty fubftance.

The

The brain, according to our author, is the part that is the laſt deſtroyed.

As it is to the extrication of aeriform fluids from the dead body during putrefaction, and to the re-action of thoſe fluids on the body itſelf, that our author thinks we are to aſcribe the formation of this ſubſtance, ſo he obſerves that it is not till the ſurrounding earth is ſaturated with theſe fluids that the change begins to take place. This ſaturation of the earth he thinks is proved by its black colour. Expoſed to the air, it ſoon, he obſerves, loſes this appearance, and becomes capable of diſſolving the fatty ſubſtance in queſtion. He has found this ſubſtance only in the common pits where the ſurrounding earth has acquired this black colour; he has never been able to diſcover any traces of it in ſingle graves; he therefore concludes that an accumulation of animal bodies in large maſſes is requiſite for its formation, and alſo that theſe maſſes muſt be ſufficiently covered with earth o prevent the evaporation of the aeriform fluids that are extricated, becauſe in proportion as theſe eſcape, the ſaturation of the ſurrounding earth becomes leſs complete.

But beſides the evaporation of theſe fluids which takes place ſooner or later, another cauſe is mentioned by our author as contributing very powerfully to the deftruction of the bodies thus transformed, and that is the moifture of the foil, which by reafon of the foapy nature of the fubftance in queftion is found to diffolve it very completely. The ftate of the earth, in this refpect, is, therefore, one of the principal circumftances on which the duration of this fubftance depends. Our author accordingly obferved that in the pits the leaft expofed to the fun, and which, from their fituation in other refpects, were moft liable to moifture, the bodies were the moft fpeedily decompofed. He has even feen coffins in an inclined pofition, in one part of which, expofed to the action of moifture, the fubftance in queftion was completely diffolved, while in the dry part it had undergone no change.

Of this curious phenomenon, which feems hitherto to have efcaped obfervation, M. Thouret remarks that it adds new facts to the hiftory of the decompofition of animal bodies in the earth, and may be confidered as a particular fpecies of mummyfication, which, compared with that which produces the dry and fibrous mummy, fhews us in this way a new procefs of nature. Both thefe fpecies of mummy, he obferves, depend on the action of aeriform fluids.

Thus the deſtruction of the body takes place if theſe evaporate; the ſpecies of mummy, which is more immediately the ſubject of this paper, is produced if theſe fluids when diſengaged are reflected on the ſoft parts of the body or retained in their texture; and, on the other hand, the dry and fibrous mummy is formed whenever theſe ſame fluids are not at all or imperfectly diſengaged.

On ſimilar principles, he thinks, may be explained the different circumſtances obſerved in the decompoſition of bodies in burial grounds, whether in ſeparate or in common graves; thoſe circumſtances more eſpecially which may be aſcribed to the nature of the ſoil. In general, he obſerves, they will depend on the facility with which it abſorbs or tranſmits the different ſpecies of air extricated from bodies by putrefaction, and hence, dry ſand is, he thinks, the moſt favorable to the decompoſition of bodies. This decompoſition will alſo, he adds, be accelerated by calcareous earths, which are known to be very porous and permeable, and for this reaſon have been called putrid or ſeptic earths. On the other hand, compact argillaceous earths, he obſerves, are found to retard this decompoſition, as was mentioned by Meſſieurs Lemery,

Geoffroy, and Hunauld, in their report to the Academy of Sciences in 1738.

Thefe facts, the author farther remarks, ferve to fhow how little foundation there is for the opinion commonly entertained relative to the converfion of the dead body into earth, no fuch appearance having been obferved in any of the coffins that were intire; neither, he adds, is what is ufually imagined true that the body is, in general, deftroyed by worms, as thefe are found only near the furface of the earth, or in bodies that have been expofed to the air. His obfervations have convinced him that human bodies configned to the earth infenfibly exhale and evaporate in volatile principles; and for this reafon it is, he thinks, that the foil of burial places does not perceptibly accumulate.

M. Thouret preferves in his collection fpecimens procured in thefe refearches, and which ferve to confirm and illuftrate all the facts he has related in the report before us; and he propofes in fubfequent papers to defcribe the different parts of the fubject more fully, and to give engravings of the appearance of different parts of the bodies he has examined.

